# Prediction of Nontrivial Topological Phases and Rashba Spin-Splitting in BaABTe_4_ Janus Monolayers (A, B = Al, Ga, In, or Tl)

**DOI:** 10.1021/acsomega.4c11092

**Published:** 2025-04-10

**Authors:** Joel D’Souza, Rovi Angelo B. Villaos, Aniceto B. Maghirang III, Ina Marie R. Verzola, Sreeparvathy Puthiya Covilakam, Zhi-Quan Huang, Feng-Chuan Chuang

**Affiliations:** † Department of Physics, National Sun Yat-sen University, Kaohsiung 80424, Taiwan; ‡ Physics Division, National Center for Theoretical Sciences, Taipei 10617, Taiwan; § Center for Theoretical and Computational Physics, National Sun Yat-sen University, Kaohsiung 80424, Taiwan; ∥ Department of Physics, National Tsing Hua University, Hsinchu 30013, Taiwan

## Abstract

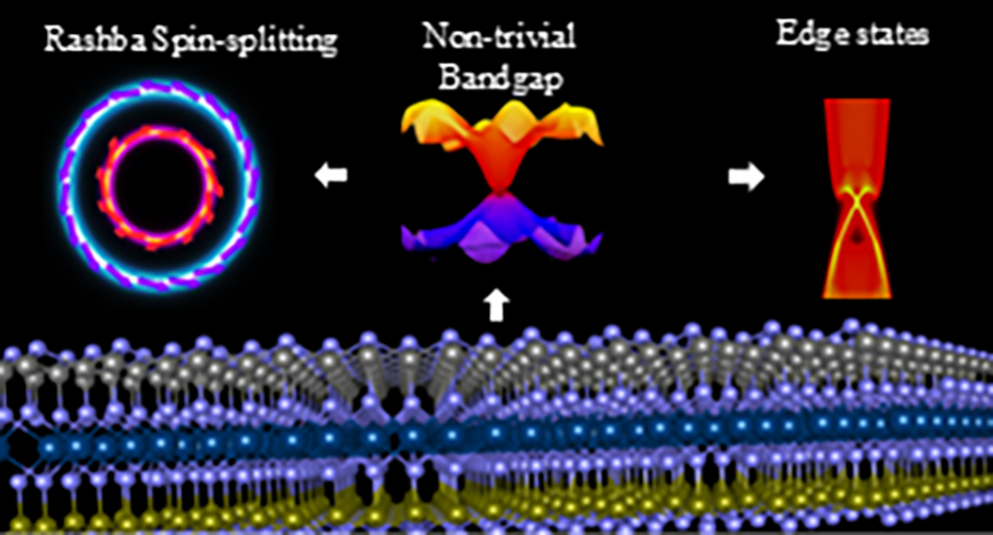

Two-dimensional (2D) materials have emerged as a significant focus in materials research due to their tunable properties on thermoelectricity, spin-splitting, and nontrivial topology. Specifically, Janus-type 2D materials are interesting due to their additional breaking of inversion or mirror symmetry in the atomic structure. Based on the recently synthesized monolayer MoSi_2_N_4_ and previously studied BaIn_2_Te_4_ with the chemical formula of MA_2_Z_4_, we derive a family of 2D Janus compounds, namely BaABTe_4_. Using first-principles calculations, a total of six Janus BaABTe_4_ monolayers (BaAlGaTe_4_, BaAlInTe_4_, BaAlTlTe_4_, BaGaInTe_4_, BaGaTlTe_4_, and BaInTlTe_4_) were investigated for their dynamical stability, electronic, and topological properties. Notably, the Z_2_ topological invariant calculated using HSE06 hybrid functional reveals that three out of the six monolayers (BaAlGaTe_4_, BaAlTlTe_4_, and BaInTlTe_4_) have nontrivial topological phases, with BaInTlTe_4_ exhibiting the largest positive system band gap of 17 meV. These three topological monolayers were further confirmed to be dynamically stable based on phonon dispersion and formation energy calculations. Subsequent orbital analysis of BaInTlTe_4_ showed that the spin–orbit coupling effect drives the topological phase transition, resulting in the band inversion between the *s*-orbital of In + Tl and *p*_*x*_ + *p*_*y*_-orbitals of Te around Γ. Also, the presence of the gapless edge states confirmed the nontrivial topological property. The Janus monolayers were found to exhibit significant Rashba spin-splitting except BaAlInTe_4_. The topologically nontrivial BaAlTlTe_4_ has the strongest Rashba strength of α^K-Γ^= α^Γ-M^ = 1.03 eVÅ. Our results show that the coexisting nature of the nontrivial phase and Rashba-type splitting within the BaABTe_4_ Janus monolayers might apply to spintronics.

## Introduction

Topological materials are an interesting class of compounds characterized by their peculiar electronic and topological properties, resulting in many fascinating phenomena and possible applications.^1−5^ In contrast to conventional materials, topological materials have a nontrivial topological order, which results in conducting edge states that are localized at the edges while the bulk of the material remains insulating.^1,6^ Further, numerous two-dimensional (2D) materials have been studied and are predicted to have nontrivial topology.^7^ Among them, graphene is the first 2D material shown to exhibit nontrivial topological properties, further expanding our understanding of 2D materials since they have characteristics that set them apart from their three-dimensional (3D) counterparts.^2^ Graphene is also well-known for its extraordinary electrical properties, including high electron mobility and good mechanical strength.^8^ Since its discovery, many novel 2D materials have been predicted with topological properties, and several have been synthesized.^9−13^ Beyond graphene, the following 2D topological materials with honeycomb structures or hexagonal lattice have been reported: III–V buckled honeycombs,^14,15^ MXenes,^9,16−19^ ternary transition metal chalcogenides,^20,21^ Janus materials,^22−25^ functionalized Bi/Sb,^26−29^ arsenene oxide,^30^ and RuClBr.^31^ Although much research has been done on identifying 2D topological materials^10,14,15,20,32^ based on honeycomb structures and a hexagonal lattice, research efforts are continuously being made to find materials with nontrivial topological characteristics.

The transition of research interest from pristine 2D materials to Janus 2D materials came into focus after the successful synthesis of 2D MoSSe from the selenization of MoSe_2_^33^ and sulfurization of MoSe_2_.^34^ The breaking of crystal symmetry can be achieved through functionalization,^35−37^ heterostructures,^38,39^ or substitution^22,36^ which can lead to the Rashba effect.^40,41^ This Rashba effect induces a momentum-dependent spin-splitting in the electronic structure of 2D materials that possess asymmetry.^25^

In addition, a new family of 2D materials was recently realized by sandwiching an MN_2_ (M = Mo, Si) layer between two Si–N layers, which resulted in N–Si–N-M–N–Si-N layered structure^42^ forming the septuple layered MoSi_2_N_4_ and WSi_2_N_4_. Similarly, this 2D MA_2_Z_4_ makes constructing a 2D Janus material possible by replacing element A in MA_2_Z_4_. These Janus MA_2_Z_4_ materials have been reported to exhibit various properties, such as Rashba-type spin-splitting,^43^ valley spin-splitting,^44^ and high tunability.^45^ Very recently, Ba-based compounds have been studied experimentally^46^ and theoretically.^47,48^ Therefore, in this study, we have constructed a 2D Janus BaABTe_4_ monolayer based on the previously studied BaA_2_Z_4_ pristine monolayer.^47^ Moreover, the spin–orbit coupling (SOC) strength is one of the key factors affecting the Rashba parameter.^49^ The strength of SOC is influenced by the atomic number, with previous studies confirming that SOC increases as the atomic number of the chalcogen element rises.^50^ In our case, all the monolayers contain Te, which has a high atomic number and strong SOC. Additionally, in Janus monolayers of the MA_2_Z_4_ type, electrons can move between adjacent layers, creating a localized electric field within these layers.^51^ The nature of this charge transfer varies due to differences in atomic size and electronegativity on either side of the central Ba atom, leading to an asymmetric charge distribution in the upward and downward directions. Consequently, charge transfer is one of the variables that influences the Rashba parameter.

Moreover, the interplay between topology and Rashba-type spin–orbit coupling (SOC) remains an area of active interest, as it offers new avenues for spintronic applications beyond traditional quantum spin Hall insulators (QSHIs).^52,53^ These 2D materials with inversion asymmetry are important for a wide variety of spin transistors since they employ different operating principles based on their spin properties.^54,55^ A notable example is the Datta-Das spin transistor, also known as spin FET,^56^ which demonstrates practical applications of materials with Rashba spin-splitting. Furthermore, TIs show remarkably efficient spin-charge interconversion^52,53^ at the edge which is beneficial in storing information^57^ and attaining high processing speed.^7^ Notably, the interplay between nontrivial topology and Rashba spin-splitting creates conditions that can host Majorana quasiparticles,^58^ which play a major role in quantum computation.^59,60^

The objective of this paper is to systematically study the structural, electronic, and topological properties of six 2D Janus BaABTe_4_ monolayers using the first-principles calculations. The geometric configuration of the six Janus BaABTe_4_ (A, B = Al, Ga, In, or Tl) monolayers considered in this study is based on the stable T-phase MA_2_Z_4_ structure (space group *P*3*m*1, No. 164).^47^ The phonon dispersion and formation energy calculations showed that four materials (BaAlGaTe_4_, BaAlInTe_4_, BaAlTlTe_4,_ and BaInTlTe4) are dynamically stable. Interestingly, the stable Janus BaAlGaTe_4_, BaAlTlTe_4_, and BaInTlTe_4_ monolayers exhibit nontrivial topological properties with gapless edge states, while the breaking of inversion symmetry reveals a Rashba spin-splitting in BaAlTlTe_4_.

## Computational Methods

The first-principles calculation based on the density functional theory (DFT)^61^ was conducted using the Vienna Ab initio Simulation Package (VASP).^62,63^ The exchange-correlation effects with a cutoff energy of 400 eV were treated using projector augmented wave (PAW)^64,65^ and generalized gradient approximation in Perdew–Burke–Ernzerhof (GGA-PBE).^66−68^ A vacuum layer of 15.0 Å was inserted along the *z*-direction to prevent interactions among the repeated monolayers. Additionally, the energy convergence criterion was set to 10^–6^ eV and the monolayer structure was optimized until the residual force acting on each atom was less than 10^–4^ eV/Å. To sample the Brillouin zone, a Γ-centered Monkhorst–Pack^69^ grid of 18 × 18 × 1 was employed. The Heyd–Scuseria–Ernzerhof hybrid functional^70,71^ (HSE06) was employed through a reduced Γ-centered Monkhorst–Pack grid of 9 × 9 × 1 to get a more accurate band gap. The dynamic stability was established by calculating the phonon spectra for a supercell of size 5 × 5 × 1 using the PHONOPY^72^ package. Nosé-Hoover thermostat-based^73^ ab initio molecular dynamics simulations (AIMD) were done to confirm the thermal stability using a 5 × 5 × 1 supercell. The AIMD simulations were run for about 9 ps with a 1 fs time step at 300 K with a Γ-point restricted k-point sampling. The mechanical stability of the materials was calculated using the energy-strain method using VASPKIT.^74^ The topological phases were identified by calculating the Z_2_ number using the Z2 Pack Package.^75,76^ The Wannier90^77^ program was used for the construction of tight-binding Hamiltonians, and the topological edge states were obtained using the iterative Green’s function using the WannierTools^78^ package. The PyProcar^79^ package with a mesh grid of 21 × 21 was used to compute the spin textures. The spin–orbit coupling (SOC) effects were considered in the electronic and topological calculations.

## Results and Discussion

### Atomic Structures and Stabilities

**Figure 1:**
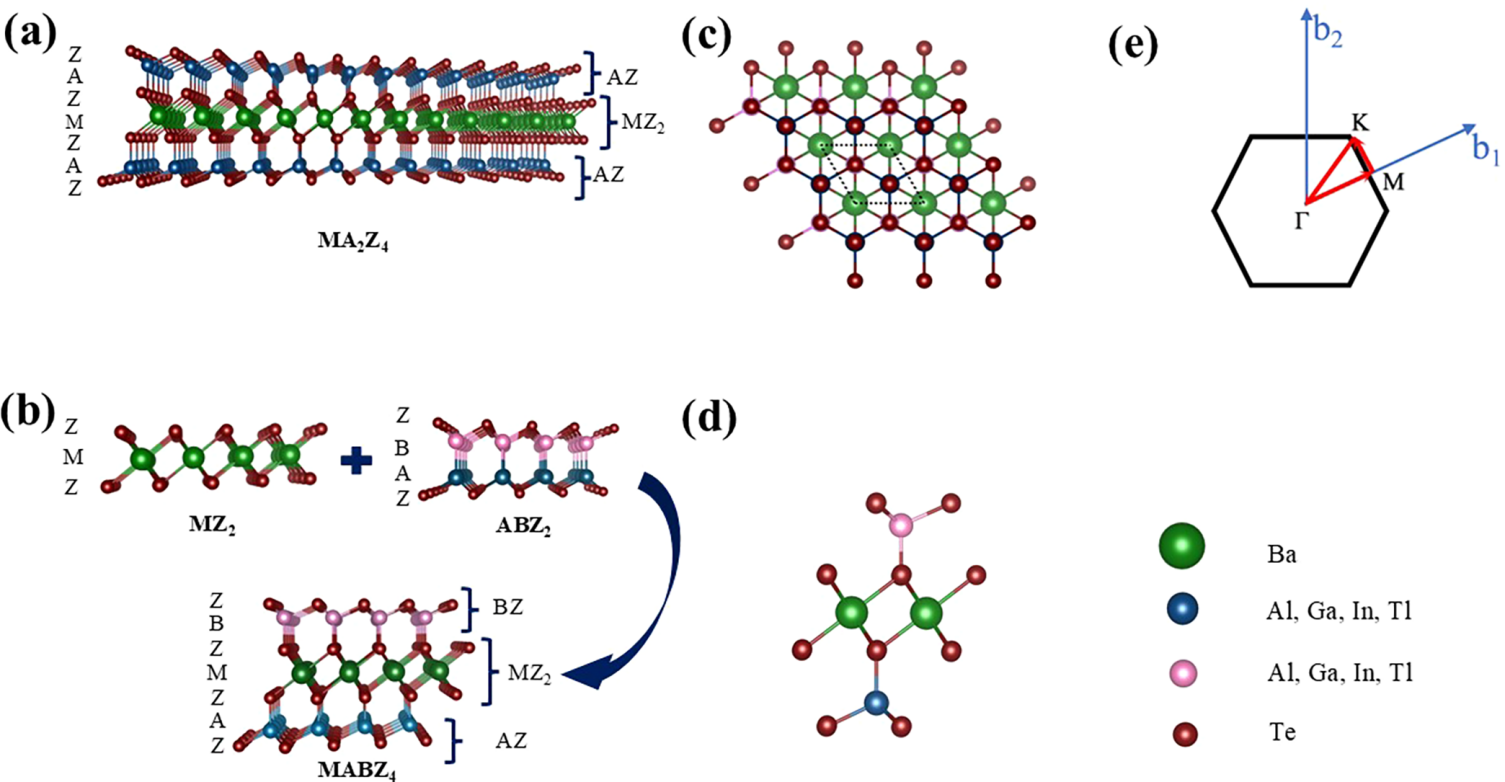
Crystal structure and first Brillouin zone of Janus MABZ_4_. Schematic representation of (a) pristine MA_2_Z_4_ and the derivative (b) Janus BaABTe_4_. The (c) top view and (d) side view of the Janus structure. (e) First Brillouin zone (BZ) of the unit cell with the high-symmetry points labeled.

The T-phase MA_2_Z_4_ was constructed by intercalating the 1T-type (MZ_2_) monolayer between two AZ layers. The resulting structure with seven atomic layers has a stacking order of Z-A-Z-M-Z-A-Z, as shown in Figure 1(a). Further, we have constructed a Janus structure by replacing A atoms with B atoms of one of the A layers in the MA_2_Z_4_ monolayer, which results in Janus MABZ_4_ monolayer with a stacking order of Z-A-Z-M-Z-B-Z as shown in Figure 1(b). Furthermore, the top view and side view of the Janus monolayer are shown in Figure 1(c,d), respectively. The first Brillouin zone (BZ) with high-symmetry points labeled is shown in Figure 1(e). To systematically study the six Janus MABZ_4_ monolayers, we first performed geometric optimization under GGA-PBE. The optimized lattice parameters for all the materials are summarized in Table 1. The dynamic stability was then examined via phonon dispersion calculations. The phonon dispersion spectra of the monolayers calculated along the high-symmetry points of the BZ are shown in Figure S1. The phonon dispersion curves show no imaginary frequencies in BaAlGaTe_4_, BaAlInTe_4_, BaAlTlTe_4_, and BaInTlTe_4,_ indicating that these monolayers are dynamically stable. Also, from the calculation of formation energies per formula unit (Tables S1 and S2), it can be seen that the Janus structures (inversion symmetry-broken system) and pristine structures (inversion symmetric system) have comparable values. This implies that the Janus structures are experimentally as viable as their pristine counterparts. Notably, the formation energies of the Janus structures like BaAlGaTe_4_ (−5.116 eV) are within the range of their pristine counterparts, BaAl_2_Te_4_ (−6.041 eV) and BaGa_2_Te_4_ (−3.930 eV), further reinforcing their stability. To strengthen our stability analysis, we performed ab initio molecular dynamics (AIMD) simulations for about 9 ps, and we observed no obvious deformations in the structures (see Figure S2), which confirms the thermal stability of BaAlTlTe_4_ and BaInTlTe_4_. Additionally, we assessed the mechanical stability of these monolayers using their elastic constants *C_ij_*. Given the hexagonal symmetry, the independent elastic constants were found to be *C*_11_ = *C*_22_ = 83 N m^1–^ (BaInTlTe_4_)/ 74 N m^1–^ (BaAlTlTe_4_) and *C*_12_ = 24 N m^1–^ (BaInTlTe_4_)/21 N m^1–^ (BaInTlTe_4_), the Born criteria of stability^80^ is *C*_11_ > 0, *C*_66_ > 0. Here, *C*_66_ is (*C*_11_–*C*_12_)/2. Therefore, the calculated *C_ij_* confirms the mechanical stability of BaAlTlTe_4_ and BaInTlTe_4_.

**Table 1:** Calculated System Band Gap, Band Gap at Γ, and Topological *Z*_2_ Invariant Number of Janus BaABTe_4_ Monolayers Using HSE06^a^

Material	Lattice parameter, *a = b* (Å)	System band gap (meV)	Band gap at Γ (meV)	*Z*_2_ invariant number	Rashba parameter (eVÅ) (α_R_^K-Γ^) = (α_R_^Γ-M^)
BaAlGaTe_4_	4.430	16	63	1	0.21
BaAlInTe_4_	4.218	302	302	0	0.00
BaAlTlTe_4_	4.218	–28	24	1	1.03
BaGaInTe_4_	4.219	26	26	0	0.95
BaGaTlTe_4_	4.220	26	161	0	0.71
BaInTlTe_4_	4.689	17	168	1	0.09

a The negative system band gap denotes that the top of the valence band is higher than the bottom of the conduction band in energy. The trivial phases are indicated by *Z*_2_ = 0, while the nontrivial phases are *Z*_2_ = 1.

### Electronic and Topological Properties of BaInTlTe_4_

To examine the electronic properties of the Janus MABZ_4_ monolayers, the electronic band structures were calculated using GGA-PBE, shown in Figures S3 and S4, while the band gaps are summarized in Table S3. To study their topological properties, the *Z*_2_ topological invariant number was calculated. A material with *Z*_2_ = 1 exhibits a nontrivial topological phase, while a material with *Z*_2_ = 0 represents a conventional or trivial material. Interestingly, five out of the six compounds (BaAlGaTe_4_, BaAlTlTe_4_, BaGaInTe_4_, BaGaTlTe_4_, BaInTlTe_4_) were found to be topologically nontrivial under GGA-PBE based on the calculated Z_2_ number equal to 1, as summarized in Table S1.

Since GGA-PBE is known to underestimate the band gaps,^70,81^ a hybrid functional approach (HSE06) was further employed for high-accuracy prediction of the band gaps. The band structures obtained under HSE06 of all six compounds are shown in Figure S5, and their corresponding system band gaps and band gaps at Γ are summarized in Table 1. The band structures with spin–orbit coupling (SOC) indicate that BaAlGaTe_4_, BaAlInTe_4_, BaGaInTe_4_, BaGaTlTe_4_, and BaInTlTe_4_ monolayers are insulating under the hybrid functional approach, with narrow system band gaps of 16, 302, 26, 26, and 17 meV, respectively. On the other hand, the BaAlTlTe_4_ monolayer has a negative system band gap. Furthermore, we recalculated the Z_2_ number of all six Janus materials under HSE06. Remarkably, three materials (BaAlGaTe_4_, BaAlTlTe_4_, BaInTlTe_4_) retained their *Z*_2_ values to be 1 and thus identified as topologically nontrivial, as summarized in Table 1.

**Figure 2:**
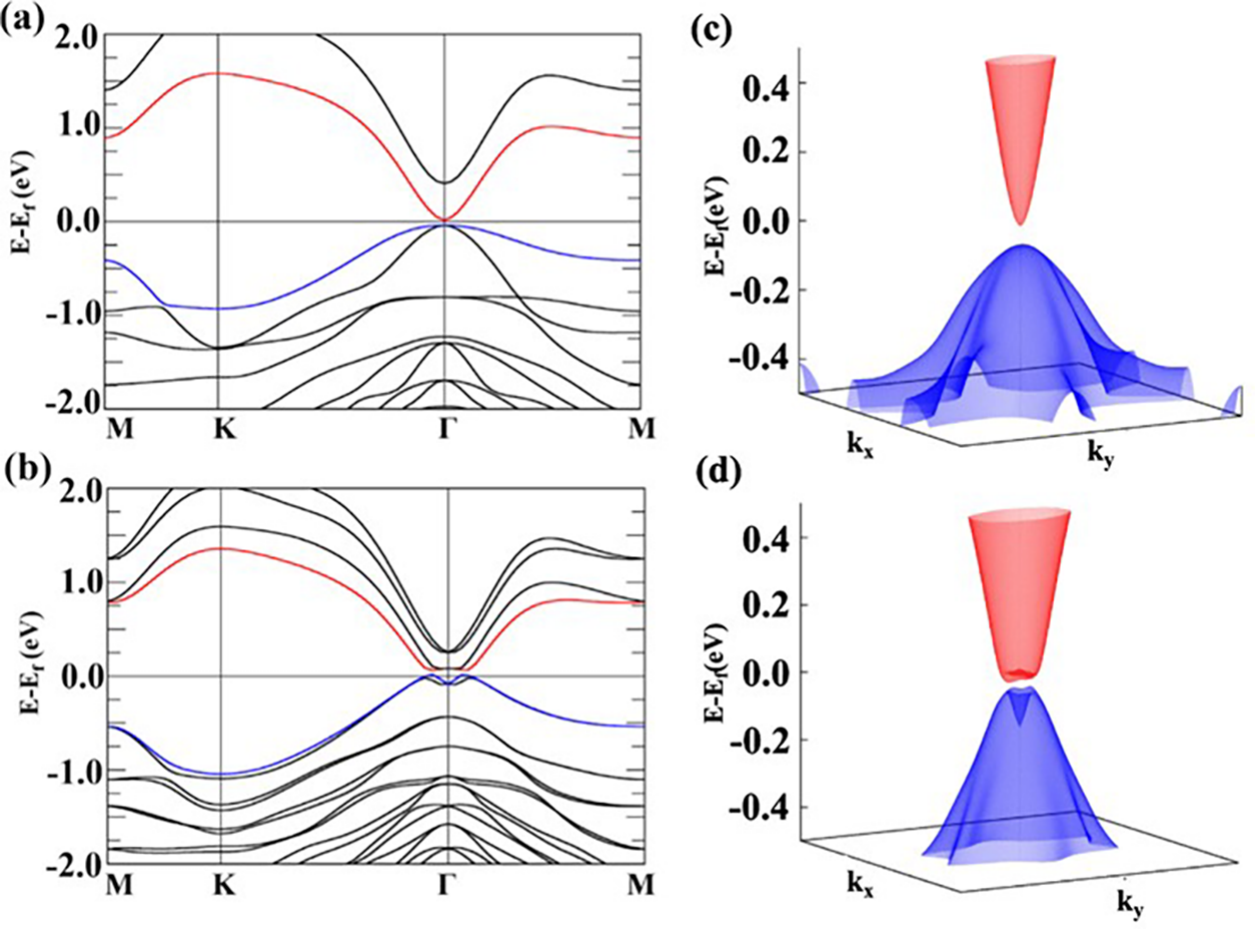
Band structures for BaInTlTe_4_ using the HSE06 functional (a) without SOC and (b) with SOC. The blue line corresponds to the valence band maximum (VBM), while the red line represents the conduction band minimum (CBM). The 3D electronic band structure under HSE06 (c) without and (d) with SOC.

To further elucidate the topological properties of these compounds, we selected BaInTlTe4 as our representative material since it has retained the *Z*_2_ number equal to 1 and has the largest band gap at Γ under the HSE06 functional. Figure 2(a,c) shows that the electronic band structure under HSE06 without SOC depicts the system as a narrow-gap insulator with a band gap of 54 meV at the Γ-point. With the inclusion of SOC, as shown in Figure 2(b,d), the band gap increased to 168 meV at the Γ-point. Interestingly, the inclusion of SOC induces a downward bending of the valence band at Γ similar to Bi_2_Se_3_, a well-known topological insulator.^82^

**Figure 3:**
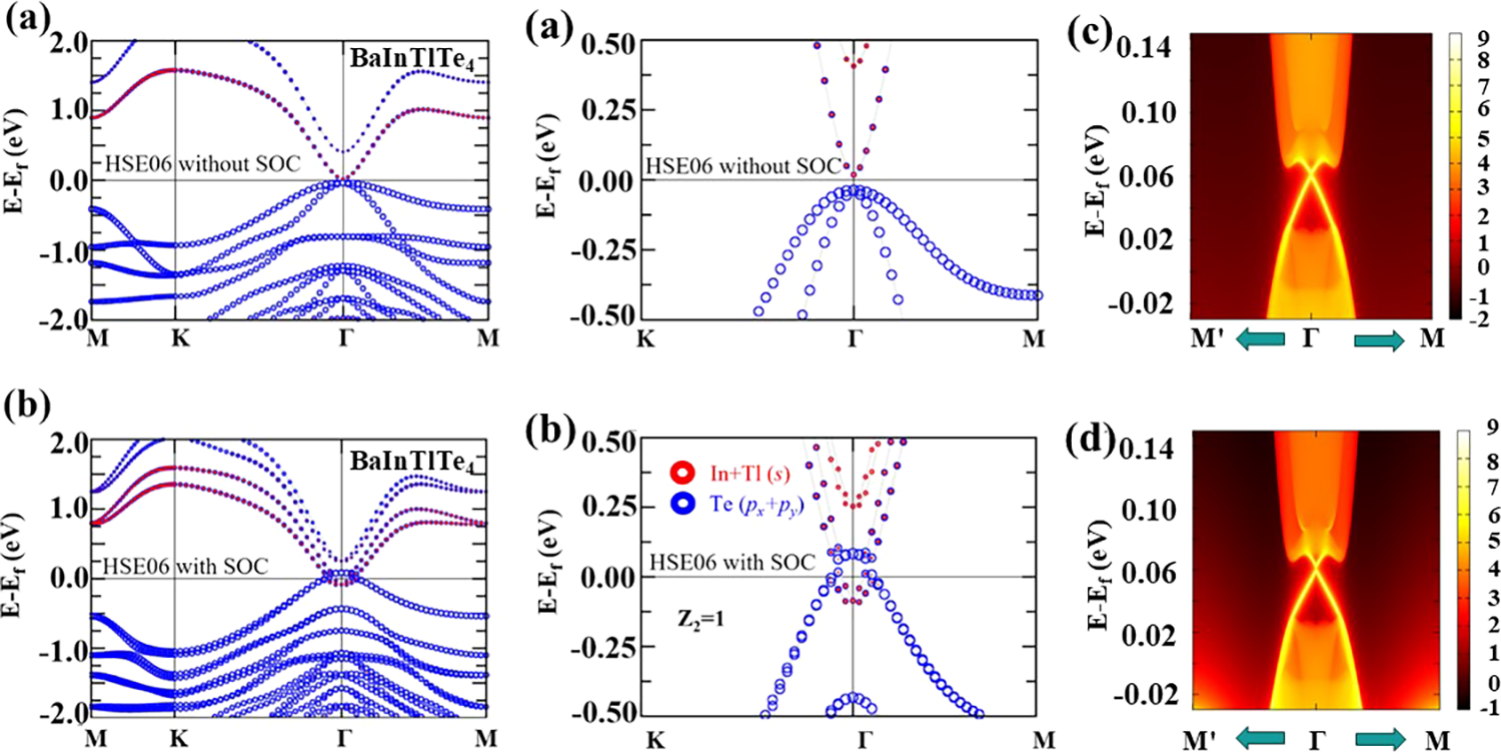
Orbital-projected band structures of monolayer BaInTlTe_4_ under HSE06 (a, c) without and (b, d) with SOC. Edge states for the monolayer BaInTlTe_4_ along (e) the left edge and (f) the right edge.

A detailed orbital analysis was then performed on BaInTlTe_4_ to further understand the mechanism behind the nontrivial topological phase. A partial band projection for BaInTlTe_4_ is shown in Figure 3(a,d), and it was found that Te-*p*_*x*_ + *p*_*y*_ (blue) and In + Tl-*s* (red) were the major contributors of orbitals near the Fermi level. Therefore, BaInTlTe_4_ has the nontrivial topological phase, which is driven by the SOC-induced band inversion near the Fermi level at Γ. Moreover, the existence of a nontrivial phase is accompanied by the edge states. Hence, for further confirmation of the topological properties, we calculated the edge states of the BaInTlTe_4_ monolayer under HSE06. Interestingly, in Figure 3(e,f), we observe a gapless edge state that spans the bulk energy gap that links the conduction and valence bands. Thus, confirming that BaInTlTe_4_ monolayer is indeed a topological insulator.

### Spin Properties of BaAlTlTe_4_

**Figure 4:**
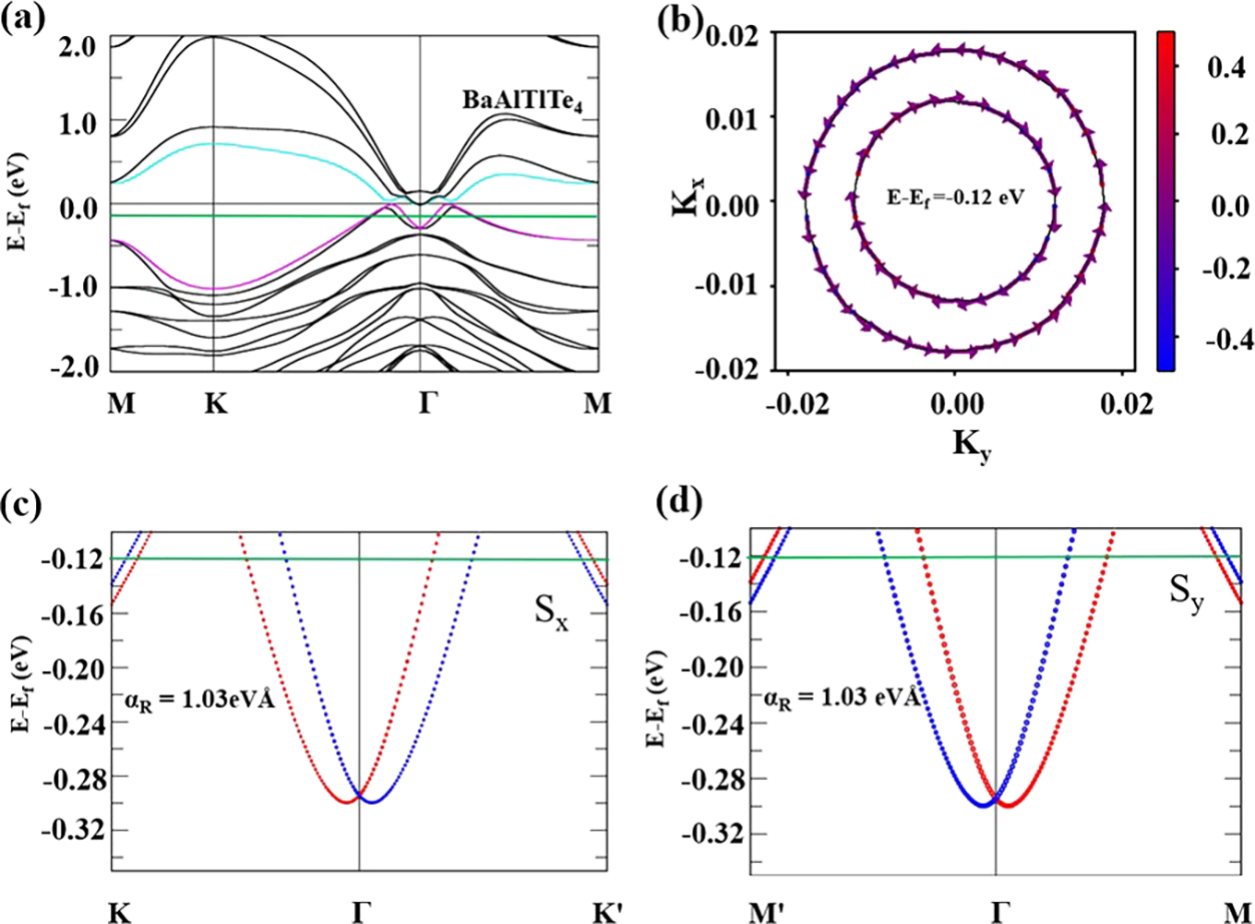
Rashba spin-splitting in 2D Janus BaAlTlTe_4_. (a) Band structure with SOC. (b) Energy contour of 2D spin textures of BaAlTlTe_4_ at *E* – *E*_f_ = −0.12 eV, respectively. The band structures along (c) K-Γ-K’ and (d) M’-Γ-M. The red and blue circles in panel (b) correspond to S_*x*_^+^ and S_*x*_^–^, and, the red and blue circles in panel (d) correspond to S_*y*_^+^ and S_*y*_^–^, respectively. The green line corresponds to the energy slicing of the 2D spin texture.

Aside from the apparent topological insulating phase in the Janus MABZ_4_ monolayers, we have also observed the existence of SOC-induced Rashba spin-splitting due to inversion symmetry breaking. As seen in Table 1, the BaAlTlTe_4_ monolayer has the largest Rashba parameter. Hence, it will be used as the representative material for further discussion on the Rashba effect. Figure 4(a) unveils the band structure under GGA-PBE with SOC. Upon careful examination of the band structures, we observed that BaAlTlTe_4_ exhibits a Rashba splitting at the VBM around the Γ-point, upon inclusion of SOC. Furthermore, it is also evident from Figure 4(a) that SOC mediates a band splitting along the high-symmetry points K-Γ-M.

To further understand the spin configuration of the Rashba bands, we plotted 2D spin texture with the energy slicing of *E* – *E*_F_= −0.12 eV. The green dotted line in Figure 4(a–d) represents the energy level corresponding to the slicing. The in-plane vector components S_*x*_ and S_*y*_ are represented by the arrows, and we can see that there exists clockwise and counterclockwise spin rotation, which is a characteristic of the Rashba effect. In contrast, the red–blue color gradient shows the S_*z*_ vector component, which is out of plane. Moreover, for the propagation of the spin, centered at the Γ-point below the Fermi-level *E*_F_, the inner band follows a clockwise rotation, whereas the outer bands follow a counterclockwise rotation as shown in Figure 4(b). As the in-plane spin components (S_*x*_, S_*y*_) have different spin orientations at the inner and outer bands, the 2D spin textures computed at Γ point below the *E*_f_ are consistent with the Rashba effect feature. Furthermore, the strength of the Rashba spin-splitting was calculated using the formula α_R_ = 2*E*_R_/*k*_R_. The calculated Rashba parameters along K-Γ-K’ (α_R_^K-Γ^) and along M’-Γ-M (α_R_^Γ-M^) are both equal to 1.03 eVÅ, showing that the splitting is isotropic.

The Rashba parameter of 1.03 eVÅ for the BaAlTlTe_4_ monolayer is significant, positioning it between well-known monolayers such as BiTeBr (1.34 eVÅ),^83^ SnTe (0.88 eVÅ),^83^ and PtSSe (1.654 eVÅ),^25^ and is considerably larger than that of InSnCl_3_ (0.27 eVÅ),^83^ and PtSeTe (0.435 eVÅ).^25^ This substantial Rashba-like splitting makes it a promising candidate for spintronic applications.

With these interesting properties of BaABTe_4_ Janus monolayers (A, B = Al, Ga, In, or Tl), it is crucial to verify these findings experimentally. We now propose a potential experimental strategy for experimental synthesis. Chemical vapor deposition (CVD) can be used to synthesize the pristine BaA_2_Te_4_ monolayer (Te-A–Te–Ba-Te-A-Te), similar to the already experimentally synthesized MoSi_2_N_4_.^42^ Then, to create the Janus structure, the top two layers could be stripped off using a hydrogen plasma. Without disrupting the vacuum, using a flowing hot gas of B–Te could replace the hydrogen and yield BaABTe_4_ Janus monolayers (Te-A–Te–Ba-Te–B-Te).^24,84^ Indeed, the calculated phonon dispersion and the formation energies of the Janus monolayers show that these materials are dynamically stable and could be synthesized. For the verification of the synthesized BaABTe_4_ monolayers, the thickness of the synthesized material could be determined using atomic force microscopy (AFM)^42^ and the characterization of the material could be done using Raman spectroscopy.^24^

## Conclusions

In conclusion, we investigated a family of Janus-type 2D materials, BaABTe_4_, derived from the MA_2_Z_4_ structure and demonstrated their potential for advanced applications. Using first-principles calculations, we explored the dynamical stability, electronic, and topological properties of six BaABTe_4_ monolayers. Notably, three monolayers (BaAlGaTe_4_, BaAlTlTe_4_, and BaInTlTe_4_) exhibited nontrivial topological phases, with BaInTlTe_4_ displaying the largest positive band gap of 17 meV. Dynamical stability was confirmed through phonon dispersion, while orbital analysis revealed that spin–orbit coupling drives the topological phase transition via band inversion between the *s*-orbitals of In+Tl and the *p*_*x*_*+ p*_*y*_ orbitals of Te around Γ. The presence of gapless edge states further validated their nontrivial topological properties. Most Janus monolayers demonstrated significant Rashba spin-splitting, with BaAlTlTe_4_ exhibiting the strongest Rashba strength (α^K-Γ^ = α^Γ-M^ = 1.03 eVÅ). These findings highlight the coexistence of nontrivial topology and Rashba-type splitting in BaABTe_4_ monolayers, emphasizing their potential for spintronic applications.

## Supporting Information Available

Phonon dispersion spectra; formation energies; AIMD simulations and band structures using GGA-PBE and HSE06 (PDF)
