# Supplementary material for: Prediction of Nontrivial Topological Phases and Rashba Spin-Splitting in BaABTe4 Janus Monolayers (A, B = Al, Ga, In, or Tl)
Source: ACS Omega. 2025 Apr 10;10(15):15272–9. doi: 10.1021/acsomega.4c11092 (PMC12019431; doi:10.1021/acsomega.4c11092)
Supplement: Supplementary file 1 — ao4c11092_si_001.pdf [file ao4c11092_si_001.pdf]

Supporting Information for

Prediction of non-trivial topological phases and Rashba spin-splitting in $BaABTe_4$ Janus monolayers (A, B = Al, Ga, In, or Tl)

Joel D'Souza[1] , Rovi Angelo B. Villaos[1], Aniceto B. Maghirang III[1], Ina Marie R. Verzola[1], Sreeparvathy Puthiya Covilakam[1,2], Zhi-Quan Huang[1], and Feng-Chuan Chuang[1,2,3,4*]

[1]*Department of Physics, National Sun Yat-sen University, Kaohsiung, 80424 Taiwan*

[2]*Physics Division, National Center for Theoretical Sciences, Taipei, 10617 Taiwan*

[3]*Center for Theoretical and Computational Physics, National Sun Yat-sen University, Kaohsiung, 80424 Taiwan*

[4]*Department of Physics, National Tsing Hua University, Hsinchu, 30013 Taiwan*

*Corresponding Author: Feng-Chuan Chuang

Postal Address: 70 Lienhai Rd., Kaohsiung 80424, Taiwan.

Telephone: +886-7-5253733

E-mail Address: fchuang@mail.nsysu.edu.tw

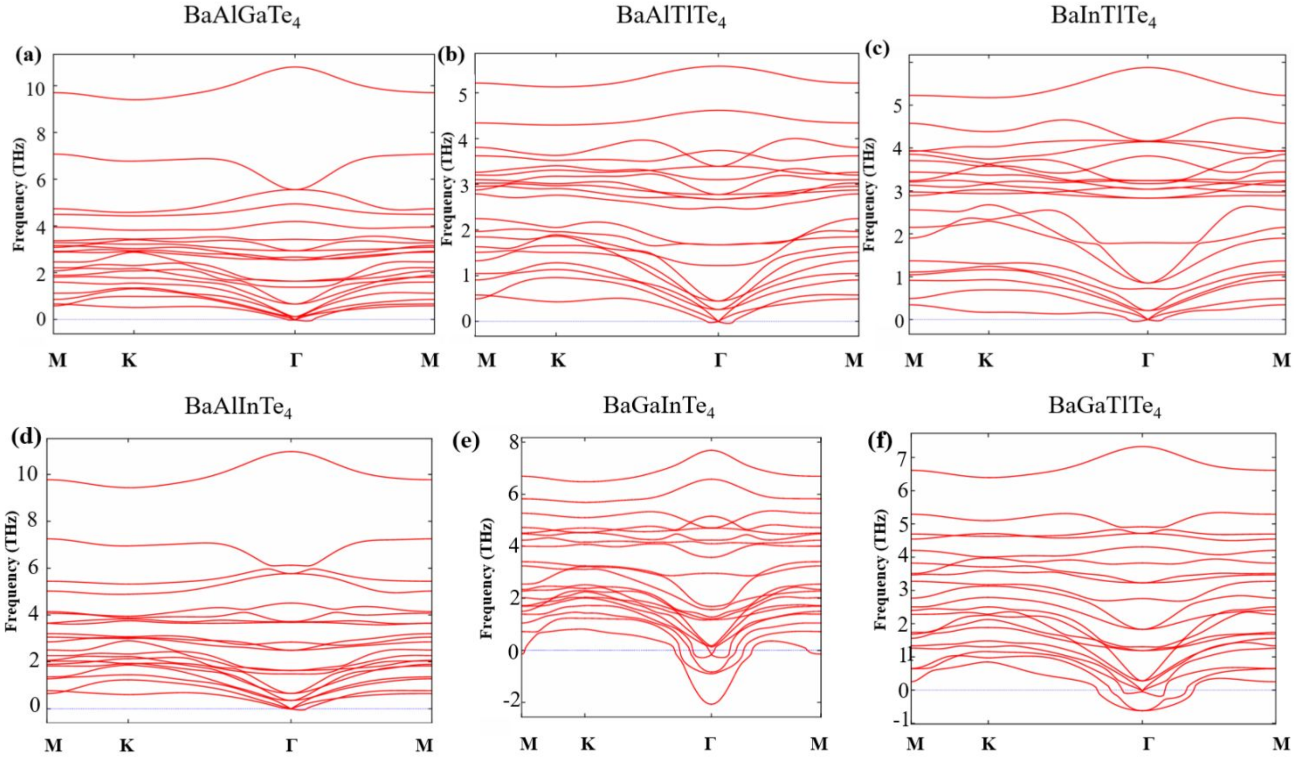

Figure S1. Phonon dispersion spectra of (a) $BaAlGaTe_4$, (b) $BaAlTlTe_4$, (c) $BaInTlTe_4$, (d) $BaAlInTe_4$, (e) $BaGaInTe_4$, and (f) $BaGaTlTe_4$ monolayers.

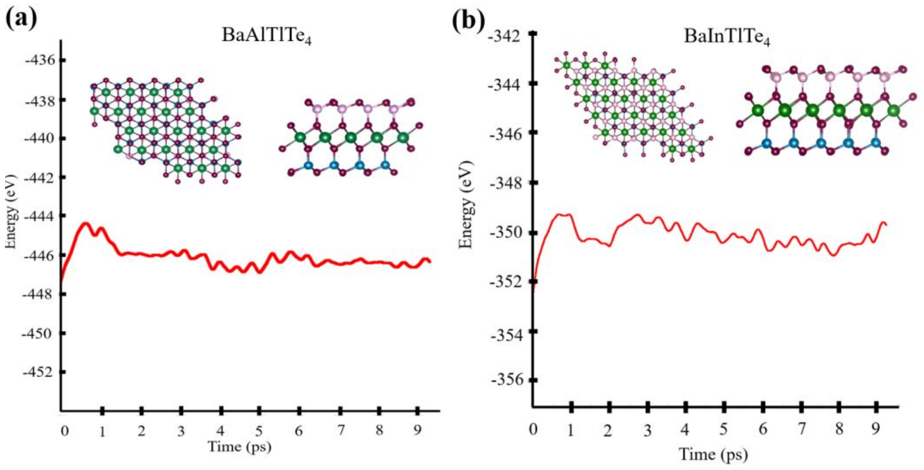

Figure S2. Total energy fluctuation with time during the AIMD simulation of (a) $BaAlTlTe_4$ and (b) $BaInTlTe_4$ at 300 K. The top and side views of the structure during the simulation are indicated as the inset

To further support the structural stability of the Janus and pristine compounds, their formation energies E_{form}(Janus) and E_{form}(pristine) for Janus and pristine cases, respectively, were calculated via the equations:

$$E_{form}(\text{Janus}) = E_{BaABTe_4} - (E_{Ba} + E_A + E_B + 4E_{Te}) \quad \text{(Eq. 1)}$$

$$E_{form}(\text{pristine}) = E_{BaA_2Te_4} - (E_{Ba} + 2E_A + 4E_{Te}), \quad \text{(Eq. 2)}$$

where A, B = Al, Ga, or In. E_{BaABTe_4} and $E_{BaA_2Te_4}$ are the total energies of the Janus ($BaABTe_4$) and pristine (BaA_2Te_4)compounds, respectively. E_{Ba}, E_A, E_B, and E_{Te} are the chemical potentials of Ba, A, B, and Te atoms, respectively, in the bulk phase.

Table S1. The formation energies per formula unit of Janus structures.

**Janus Structures**	
**Materials**	**Formation energy per formula unit (eV)**
$BaAlGaTe_4$	-5.1161
$BaAlInTe_4$	-5.5923
$BaAlTlTe_4$	-4.2229
$BaGaInTe_4$	-4.7080
$BaGaTlTe_4$	-3.3580
$BaInTlTe_4$	-3.9605

Table S2. The formation energies per formula unit of pristine structures.

**Pristine Structures**	
**Materials**	**Formation energy per formula unit (eV)**
$BaAl_2Te_4$	-6.0418
$BaGa_2Te_4$	-3.9309

$BaIn_2Te_4$	-5.2839
$BaTl_2Te_4$	-4.6545

Table S3. Calculated system band gap, band gap at Γ, and topological Z_2 invariant number of Janus $BaABTe_4$ monolayers using GGA-PBE. The negative system band gap denotes that the top of the valence band is higher than the bottom of the conduction band in energy. The trivial phases are indicated by $Z_2 = 0$ while the non-trivial phases are $Z_2 = 1$.

Material	System Band Gap (meV)	Band Gap at Γ (meV)	Z_2 invariant number
$BaAlGaTe_4$	5	452	1
$BaAlInTe_4$	60	229	0
$BaAlTlTe_4$	-262	381	1
$BaGaInTe_4$	-91	478	1
$BaGaTlTe_4$	-268	438	1
$BaInTlTe_4$	-119	209	1

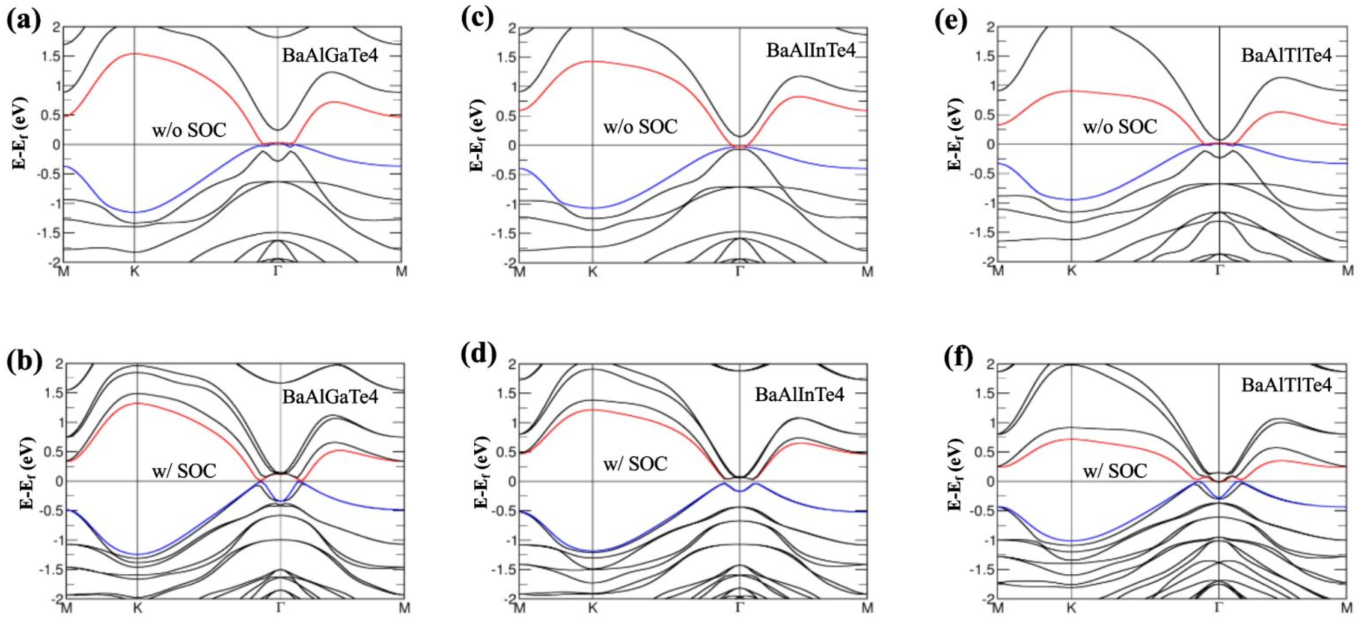

Figure S3. Band structures of $BaAlGaTe_4$, $BaAlInTe_4$, and $BaAlTlTe_4$ under GGA-PBE (a, c, and e) without SOC and (b, d, and f) with SOC.

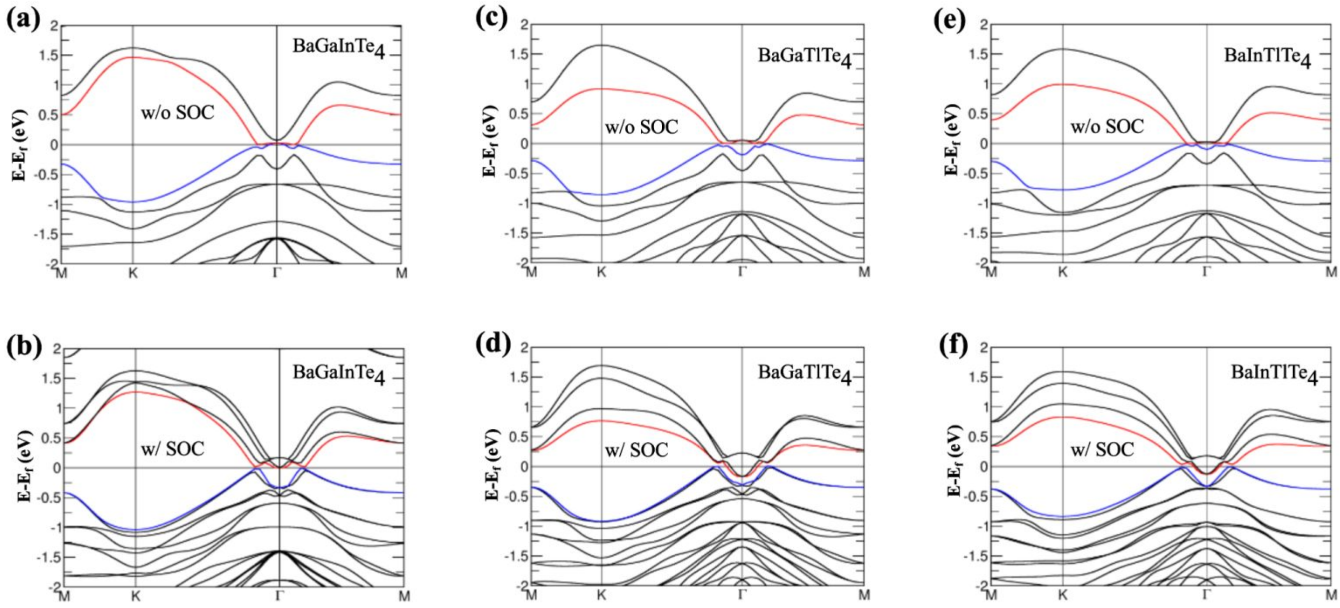

Figure S4. Band structures of $BaGaInTe_4$, $BaGaTlTe_4$, and $BaInTlTe_4$ under GGA-PBE (a, c, and e) without SOC and (b, d, and f) with SOC.

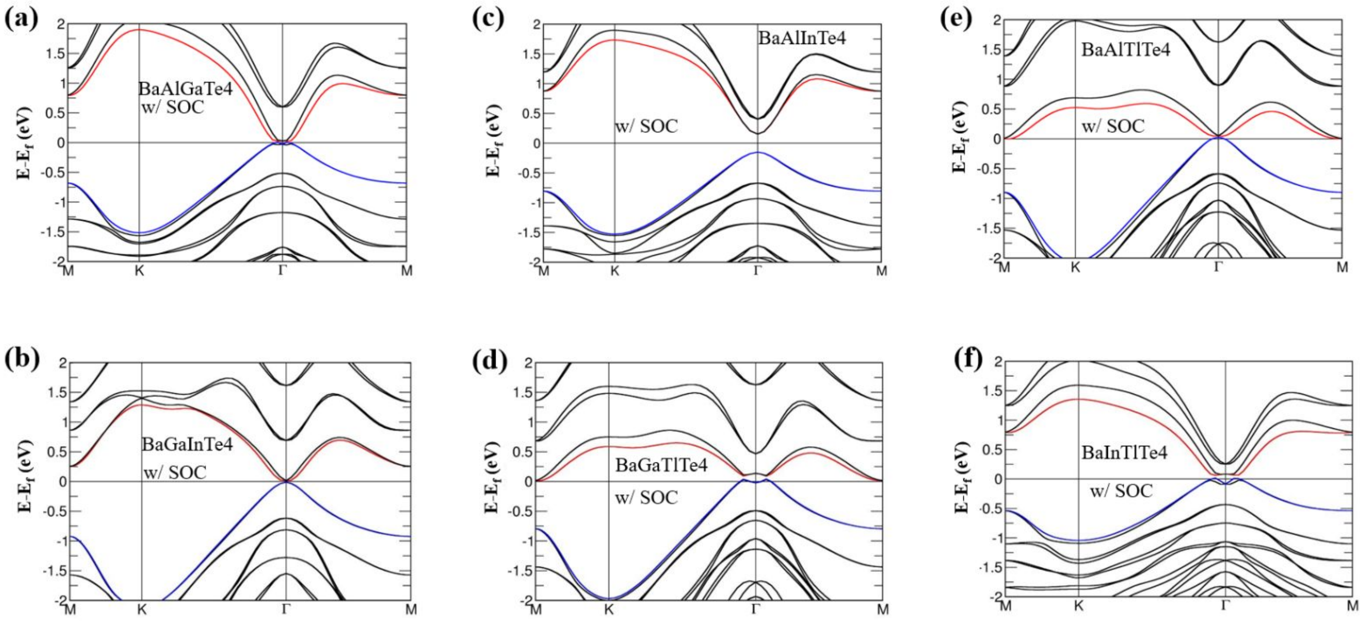

Figure S5. Band structures of (a) $BaAlGaTe_4$, (b) $BaGaInTe_4$, (c) $BaAlInTe_4$, (d) $BaGaTlTe_4$, (e) $BaAlTlTe_4$, and (f) $BaInTlTe_4$ under HSE06 with SOC.

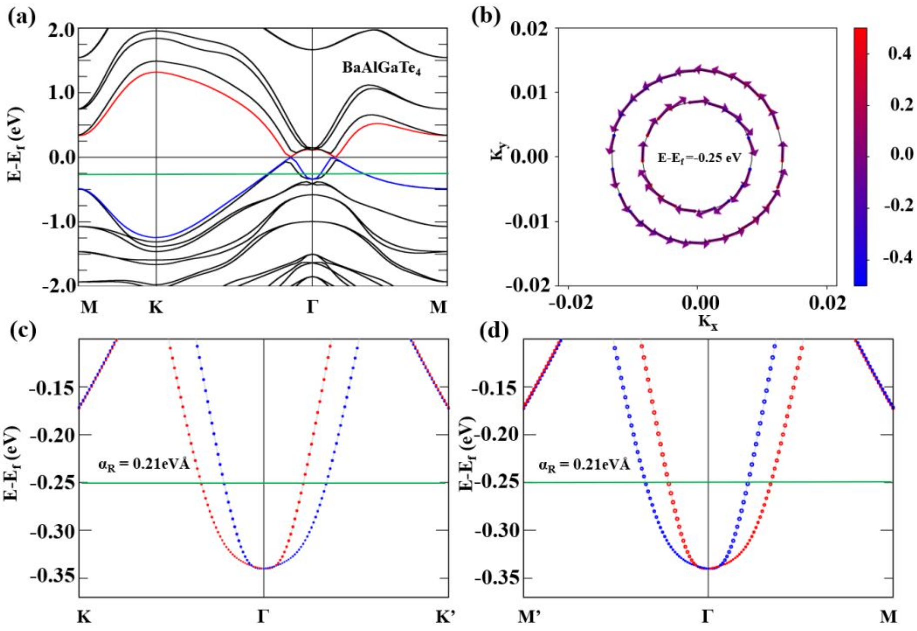

Figure S6. Rashba spin-splitting in 2D Janus $BaAlGaTe_4$. (a) The band structure with SOC. (b) The energy contour of 2D spin-textures of $BaAlTlTe_4$ at $E\text{-}E_f$ = -0.25eV. The red and blue circles of (b) correspond to S_x^+ and S_x^- respectively. The bandstructures along (c) K-Γ-K' and (d) M'-Γ-M. The red and blue circles of (d) correspond to S_y^+ and S_y^-, respectively. The green dotted line corresponds to the energy slicing of the 2D spin texture.

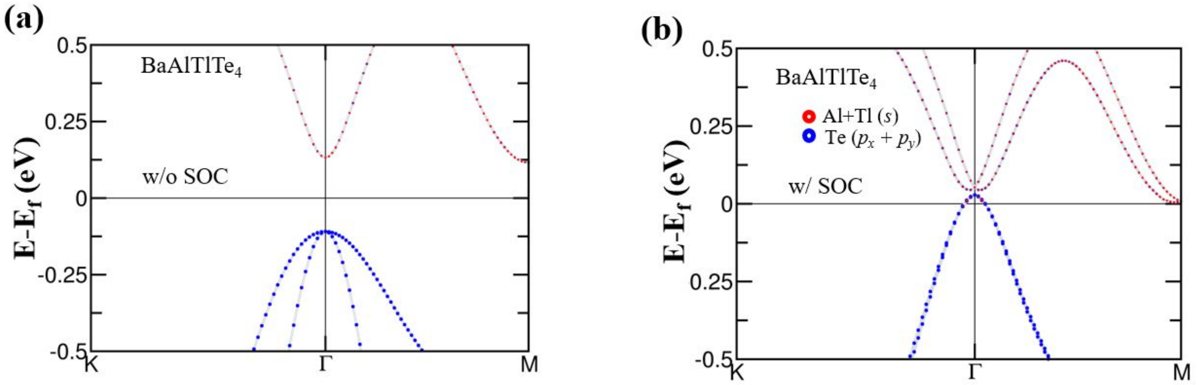

Figure S7. Orbital-projected band structures of monolayer $BaAlTlTe_4$ under HSE06 (a) without and (b) with SOC
